# Laparoscopic Unilateral Total and Contralateral Subtotal Adrenalectomy for Bilateral Adrenocorticotropic Hormone-Secreting Pheochromocytoma: Report of a Rare Case

**DOI:** 10.1089/cren.2016.0122

**Published:** 2016-12-01

**Authors:** Masanari Fukasawa, Norifumi Sawada, Tatsuya Miyamoto, Satoru Kira, Tadashi Aoki, Hidenori Zakoji, Takahiko Mitsui, Masayuki Takeda

**Affiliations:** Department of Urology, Interdisciplinary Graduate School of Medicine, University of Yamanashi, Yamanashi, Japan.

**Keywords:** bilateral ACTH-secreting pheochromocytoma, CT scan, immunostaining of ACTH, laparoscopic unilateral total and contralateral subtotal adrenalectomy, iodine-123-MIBG scintigraphy

## Abstract

***Background:*** Bilateral adrenal tumors are not common in clinical practice, but are an important source of ectopic adrenocorticotropic hormone (ACTH) secretion. Standard operative management for bilateral pheochromocytomas might dictate the removal of the involved adrenal gland and the removal of the contralateral adrenal gland. We present a case of bilateral ACTH-secreting pheochromocytoma treated with staged laparoscopic unilateral total and contralateral subtotal adrenalectomy.

***Case Presentation:*** A 58-year-old male with elevated hyperglycemia and general fatigue was hospitalized for pneumonia. CT incidentally revealed bilateral adrenal tumor. Biochemical examination was significant for elevated urinary metanephrine and normetanephrines, and plasma catecholamine level. CT scan of the head, neck, thorax, and pelvis was normal. Under the clinical diagnosis of ACTH-dependent pheochromocytoma, laparoscopic right total adrenalectomy was performed. As endocrinologic examination showed residual ACTH-dependent pheochromocytoma after surgery, laparoscopic left subtotal adrenalectomy was performed. Pathology analysis revealed pheochromocytoma with stained ACTH lesions in both adrenal tumors.

***Conclusion:*** This is a rare case of ACTH-secreting bilateral pheochromocytoma effectively treated with staged laparoscopic unilateral total and contralateral subtotal adrenalectomy, in which the production of ACTH was confirmed by immunohistochemical staining.

## Introduction and Background

Pheochromocytomas present rare but treatable neuroendocrine tumors with a highly variable clinical presentation. Most common episodes are headaches, sweating, palpitations, and hypertension. The prevalence of pheochromocytoma in patients with hypertension is 0.1% to 0.6%.^1^ Bilateral adrenal tumors are not common in clinical practice, but are an important source of ectopic adrenocorticotropic hormone (ACTH) secretion. Standard operative management for bilateral pheochromocytomas might dictate the removal of the involved adrenal gland and the removal of the contralateral adrenal gland if it is clinically enlarged.^2^ It has been described that accurate preoperative diagnoses and surgical treatment can result in total cure and preserve contralateral adrenal function. This is a first report of laparoscopically treated bilateral ACTH-secreting pheochromocytoma: unilateral total and contralateral subtotal adrenalectomy.

## Presentation of Case

A 58-year-old Japanese male was admitted to our hospital with general malaise and worsening hyperglycemia. He was already having type 2 diabetes mellitus before the admission to our hospital. Blood pressure was 90/50 mm Hg with no hypotensive drugs. Laboratory investigation revealed marked hypokalemia (potassium of 1.7 mEq/L), which was normalized with intravenous potassium supplementation. Endocrinologic investigation revealed hypercortisolism with loss of diurnal variation. Serum cortisol levels were 35.3 μg/dL at 08:00 hours, 33.8 μg/dL at 15:00 hours, and 29.4 μg/dL at 23:00 hours. Plasma ACTH levels were 205.4 pg/mL at 08:00 hours, 169.5 pg/mL at 15:00 hours, and 197.5 pg/mL at 23:00 hours. A 1 mg dexamethasone overnight suppression test yielded any suppression of cortisol (morning cortisol after dexamethasone: 32.2 μg/dL). An 8 mg dexamethasone overnight suppression test also yielded any suppression of cortisol (morning cortisol after dexamethasone: 28.7 μg/dL). Selective adrenal vein sampling was not performed because of the emerging severe and life-threatening pneumocystis pneumonia. This might induce hypovolemic state in this case.

**FIG. 1.**
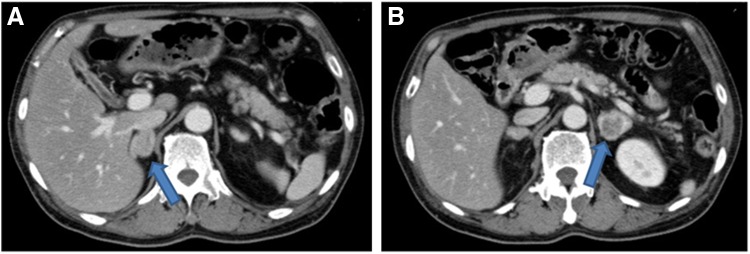
Adrenal CT scan shows right mass measuring 1.7 × 2.3 cm with mild enhancement **(A)** and a left mass measuring 2.8 × 3.3 cm with multiple areas of necrosis **(B)**.

CT scan of the abdomen revealed bilateral adrenal masses (a right mass measuring 1.7 × 2.3 cm and a left mass measuring 2.8 × 3.3 cm). Both adrenal mass lesions showed multiple areas of necrosis (Fig. 1A, B). CT scan of the head, neck, thorax, and pelvis was normal. Accumulation of the radionuclide in the right side tumor was strongly seen in iodine-123-MIBG scintigraphy. Hypokalemia, hyperglycemia, and high ACTH level suggested the existence of an ectopic source of ACTH. With biochemical investigation suggesting ACTH-driven hypercortisolemia as well as iodine-123-MIBG scintigraphy, the endocrinologists in our hospital diagnosed that the right adrenal mass was an ACTH-secreting pheochromocytoma and the left side was less functioning tumor.^3^

**FIG. 2.**
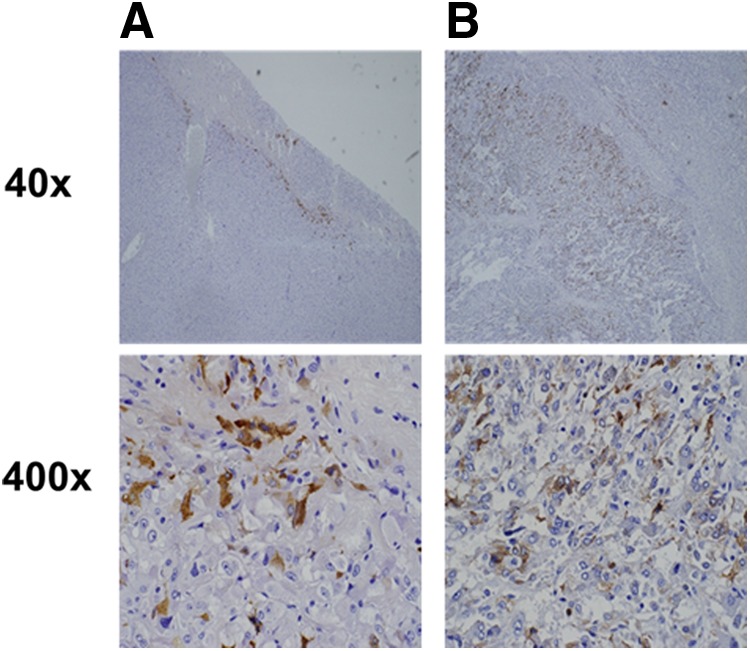
Immunohistochemical examination shows *upper panels* (40 × ) and *lower panels* (400 × ). **(A)** Right adrenal tumor immunostaining for ACTH showed partially positive. **(B)** Left adrenal tumor immunostaining was positive for ACTH in larger area.

A laparoscopic right total adrenalectomy was planned, and he was initiated on α-adrenoceptor blockade treatment (phenoxybenzamine titrated up to a dose of 1 mg/day). At the same time, the hypercortisolemia was treated with 11β-hydroxylase inhibitor metyrapone 1 gm/day. While awaiting surgery, he had suffered from pneumocystis pneumonia and was treated with trimethoprim/sulfamethoxazole. He eventually recovered from this respiratory infection and a laparoscopic right total adrenalectomy was performed. At the primary surgery, adrenal tumor with necrotic mass was found in his right adrenal gland. Histopathology analysis confirmed an adrenomedullary chromatin tumor, and immunostaining showed 5% of the tumor stained positively for ACTH (Fig. 2A). Postoperatively, his ACTH secretion and metanephrine excretion slightly decreased, but did not normalize. Therefore, we determined that there was residual ACTH-secreting lesion in the contralateral adrenal mass.

Left laparoscopic subtotal adrenalectomy was planned and he restarted taking α-adrenoceptor blockade treatment (phenoxybenzamine titrated up to a dose of 14 mg/day). Left subtotal adrenalectomy was performed with normal adrenal gland partially left. Histopathology analysis confirmed an adrenomedullary chromatin tumor, on which 10% stained positive for ACTH (Fig. 2B). After removing bilateral adrenal tumors, he had adrenal insufficiency, requiring treatment with 30 mg of oral hydrocortisone. The diurnal variation of ACTH and catecholamine was normalized. Hypokalemia and hyperglycemia resolved after these two adrenal surgeries. The histology analysis, the clinical and biochemical resolution of hypercortisolemia, and catecholamine excess finally fulfilled all of the criteria devised by Chen et al., confirming the diagnosis of an ACTH-secreting pheochromocytoma.^4^

## Discussion and Literature Review

Laparoscopic adrenalectomy has already been the standard treatment for most tumors of the adrenal gland.^2^ Patients who undergo bilateral adrenalectomy require intensive postoperative care and lifelong hormonal management. Many patients require semiurgent bilateral adrenalectomy; however, metabolic abnormalities and nutritional deficiencies should be addressed before surgery. This patient had developed severe pneumocystis pneumonia, and synchronous bilateral adrenalectomy was assessed to have higher risk for perioperative complication. The iodine-123-MIBG scintigraphy showed right ring enhancement and it suggested the primary function of the right side adenoma. From the mentioned reasons, we decided to initiate laparoscopic right total adrenalectomy. To avoid metabolic and hormonal complications associated with removal of both adrenal glands, it has been described that laparoscopic partial adrenalectomy is feasible for bilateral pheochromocytoma caused by bilateral adenomas.

Hormonal secretion did not normalize after primary surgery and contralateral laparoscopic subtotal adrenalectomy was performed. After partial resection, ACTH and cortisol levels fell to normal. Chen and colleagues established the criteria of the diagnosis of ACTH-secreting pheochromocytoma as follows: (a) clinical and laboratory evidence of hypercortisolism, (b) elevated plasma ACTH levels, (c) biochemical evidence of a pheochromocytoma (elevated urinary catecholamines, metanephrines, or vanillylmandelic acid excretion) and MRI evidence of an adrenal mass with a bright T2 signal, (d) resolution of symptoms and signs of adrenocorticoid and catecholamine excess after unilateral adrenalectomy, and (e) rapid normalization of plasma ACTH levels after adrenalectomy.^4^ Our case satisfied all of these criteria after the two-staged surgery.

Right adrenal tumor immunostaining for ACTH showed partially positive (Fig. 2A). Left adrenal tumor immunostaining was positive for ACTH in a larger lesion (Fig. 2B). Orth et al. demonstrated that the quantity of ACTH activity is correlated with the quantity of immunoreactive ACTH in tumor; however, we acknowledge limitations of immunostaining as a marker for tumorous hormone secretion and it cannot be 100% positive that the same cells had been secreting functionally hormone.

In conclusion, laparoscopically removing unilateral total and contralateral subtotal adrenalectomy for bilateral ACTH-secreting pheochromocytoma is safe and effective.

## Abbreviations Used

ACTH: adrenocorticotropin
CT: computed tomography
MIBG: metaiodobenzylguanidine
MRI: magnetic resonance imaging
